# Effect of Plant Density, Boron Nutrition and Growth Regulation on Seed Mass, Emergence and Offspring Growth Plasticity in Cotton

**DOI:** 10.1038/s41598-018-26308-5

**Published:** 2018-05-21

**Authors:** Ali Zohaib, Tahira Tabassum, Abdul Jabbar, Shakeel Ahmad Anjum, Tasawer Abbas, Azhar Mehmood, Sohail Irshad, Muhammad Kashif, Mohsin Nawaz, Naila Farooq, Irfan Rasool Nasir, Tassadduq Rasool, Mubashar Nadeem, Riaz Ahmad

**Affiliations:** 1 Department of Agronomy, University of Agriculture, Faisalabad, 38040 Pakistan; 2 Department of Agronomy, College of Agriculture, University of Sargodha, Sargodha, 40100 Pakistan; 3 Agronomy Department, University College of Agriculture and Environmental Sciences, The Islamia University of Bahawalpur, Bahawalpur, 63100 Pakistan; 4 Agricultural Training Institute, Rahim Yar Khan, 64200 Pakistan; 5 Department of Botany, University of Agriculture, Faisalabad, 38040 Pakistan; 6 Institute of Tropical Agriculture and Forestry, Hainan University, Haikou, 570228 P.R. China; 7 Institute of Soil and Environmental Sciences, University of Agriculture, Faisalabad, 38040 Pakistan; 8 Adaptive Research Farm, Dera Ghazi Khan, 32200 Pakistan

## Abstract

Seed nutrients reserves have direct relationship with seed functional traits and influence offspring performance. Effects of plant density, foliage boron (B) nutrition and mepiquat chloride (MC) growth regulation on seed nutrients reserves, seed mass and production, and emergence and offspring growth traits of cotton were studied in two years field experiment. Seed nutrients reserves and seed mass were decreased at higher maternal plant density relative to lower plant density with concomitant decrease in emergence and offspring seedling growth. However, maternal foliage B nutrition and MC growth regulation enhanced seed nutrients reserves, seed mass, emergence and offspring seedling growth performance. There was a significant positive relationship between seed mass and seed nutrients reserves indicating that changes in nutrient availability/uptake in response to maternal ecological factors determine variation in seed functional traits. Nonetheless, seed mass was positively correlated with emergence percentage and negatively with emergence timing. Furthermore, variation in offspring seedling growth traits with seed mass indicated the significance of initial seed nutrients reserves for early seedling vigour and establishment. In conclusion, lower maternal plant density, B nutrition and MC growth regulation ensued in higher emergence and offspring seedling growth of cotton because of higher seed nutrient reserves and seed mass.

## Introduction

Effects of seed size (mass) on germination and seedling establishment of perennial plants in grassland^1^ and forest^2^ ecosystems have been well understood. Seed size is correlated with various seed functional traits, and often affects the timing of germination, germination capacity, offspring seedling establishment, and diversity and composition of community^1,3,4^. Although seed mass has a great importance in natural ecosystems, its significance cannot be overwhelmed in agro-ecosystems where seed size is the primary determinant of ecosystem productivity and successful establishment of succeeding generation^5^. However, studies on the effects of ecological factors (plant competition, nutrition, precipitation, temperature, light etc.) on seed mass, subsequent germination and seedling establishment of cultivated annual crops in agro-ecosystems are sparse and not well understood^6,7^.

Cotton (*Gossypium hirsutum* L.) is cultivated as an annual crop, its perennial nature exists predominantly under environmental factors (high precipitation, temperature and nutrient inputs etc.) that favour the vegetative growth^8^. This often consequences in greater amounts of photosynthates and nutrients to be partitioned to vegetative parts while the decreased partitioning and remobilization of reserves to developing fruits become the source of variation in seed development and size^9^. Studies on natural ecosystems and perennial plants have revealed that a significant correlation exists between maternal environment and seed development and seed mass^10–12^.

Plant competitiveness, soil and plant nutrient status, and alteration in the ability of plants to acquire nutrients and their translocation to seeds can alter the pattern of seed development, seed mass, productivity and subsequent germination and seedling establishment in succeeding generation^1,2,13–15^. The plant nutrient status varies with plant species and varieties, and is determined by total nutrient supply in soil and on the factors controlling the nutrient availability to plants. Positive and regulatory effects of the seed mass are more often impossible to distinguish from those of the seed mineral nutrients reserves^16^. A greater concentration of macronutrients in surrounding ecological niche has often ensued in the production of heavier seeds having greater concentrations and reserves of N and P^17^. A recent study on perennial plant *Peucedanum oreoselinum* from Asteraceae family corroborated that a significant correlation existed between maternal habitat soil and seed macronutrients (N, P and K) contents, and seed mass and seed macronutrients contents indicating that the positive effects of seed size are more often dependent on nutrient availability and seed nutrient reserves^2^.

Plant density induces changes in the pattern of plant establishment, performance and productivity of an ecosystem, and success in the establishment of next generation because of complex relationship of inter-plant competition within a population and community^18^. An agro-ecosystem is characterized by inclusion of intensive external inputs. Thus manipulation in planting density can alter management decisions to acquire potential benefits from ecosystem services. Increasing the plant density enhances the inter-plant competition owing to limited plant available resources. Plant density modifies the plant canopy structure, plant micro environment (relative humidity, canopy temperature and light transmittance)^19^ and capability of plants to acquire mineral nutrients from the immediate rhizosphere owing to modulation in root growth due to competition for space^20^. Moreover, the seed mass is influenced due to variation in plant density. Zhi *et al*.^5^ observed higher seed number per boll with concomitant increase in seed size and vigour index at lower plant density of cotton while there was a progressive decrease in seed area (density) and vigour index with increasing plant density.

Boron is an essential micronutrient that significantly affects the seed development and quality^7,21^. A short term B deficiency during microsporogenesis hinders the development of anthers, and adversely affects the pollen viability and growth of pollen tube consequently leading to male sterility and poor seed set^21–23^. Boron nutrition affects the seed nutrients contents through modulation in uptake and translocation of other nutrients as well^24^. For example, B has positive interactions with N, P, K, Cu, Zn and Fe, whereas, negative interactions with Ca, Mg^25–27^, and Mn^28^. Moreover, it is involved in photo-assimilation and assimilate partitioning, and directly or indirectly affects the seed development and seed size^7,29^. Dordas^7^ and Dordas *et al*.^30^ observed that seed mass, yield and germination quality of cotton and sugar beets was superior in B treated plants and it was correlated with enhanced B concentration in reproductive tissues.

Exogenous application of plant growth regulators (PGRs) is a well-known strategy to improve the crop physiological performance, nutrient dynamics within plant body and crop yield under normal and stressed conditions in agro-ecosystem^8,31–33^. Some studies have provided the ground basis that exogenously applied PGRs improve the nutrient uptake and accumulation within the plant body^34,35^. Mepiquat chloride (1,1-dimethylpiperidinium chloride) is a gibberellic acid (GA) inhibitor^36^ and is used worldwide to suppress excessive vegetative growth of cotton by decreasing the plant height, internodes length, leaf area and length of fruiting and vegetative branches, and to hasten crop maturity and avoid the yield losses^37–45^. Apart from manipulation of plant canopy structure, MC enhances the cotton root growth by increasing number of lateral roots and increases the root vigor through increased reducibility and respiratory rate thereby improving the nutrient uptake and partitioning within the plant body^14,38,46,47^. Sawan *et al*.^6^ and Yang *et al*.^14^ noticed an increase in uptake and translocation of N and K to cotton seed in MC treated plants.

Effects of plant density, foliage B nutrition and MC growth regulation on seed nutrients reserves, seed traits and offspring growth performance were studied in two years multi-factor field experiment in agro-ecosystem. The objectives of the study were to (1) quantify the single and interaction effects of plant density, B nutrition and plant growth regulation on degree of variation in cotton seed nutrients reserves, seed mass and production, emergence and offspring plasticity in seedling growth (2) to elucidate relative changes in seed nutrients reserves and seed mass in response to maternal ecological factors and draw possible relationship (3) determine the quantitative relationship between seed mass and cotton seed production (4) examine the seedling emergence, emergence time and growth plasticity of cotton in next generation in relation to seed mass.

## Results

### Seed mass and seed production

The lower plant density produced greater individual seed mass of cotton as compared to higher plant density, being 3.5 and 4.6% higher during 2014 and 2015, respectively (Table 1). Foliage B nutrition enhanced the seed mass as compared to control; however, there was no significant difference between 600 mg/L and 1200 mg/L B. The heaviest seeds were produced by 1200 mg/L B nutrition, as compared to control (6.2 and 6.4% higher during 2014 and 2015, respectively) (Table 1). Likewise, single applications of MC at squaring and flowering increased the seed mass as compared to control but without significant difference between both stages of treatment. Mepiquat chloride application at squaring stage caused highest increase in seed mass (5.2 and 6% during 2014 and 2015, respectively). However, no significant interaction occurred regarding seed mass (Table 1).

**Table 1:** Individual cotton seed mass and cotton seed production as influenced by plant density, foliar boron nutrition and mepiquat chloride application.

Treatments	Individual cotton seed mass (mg)		Cotton seed production (kg ha^−1^)	
	2014	2015	2014	2015
**Density (D) (plants ha** ^**−1**^ **)**				
53333	76.3 ± 1.2 A	76.1 ± 1.1 A	1580 ± 28 B	1515 ± 25 B
88888	73.6 ± 0.7 B	72.6 ± 0.8 B	1838 ± 33 A	1665 ± 27 A
**Boron nutrition (B)**				
Control	72.5 ± 1.1 B	71.8 ± 1.1 B	1599 ± 37 C	1489 ± 28 C
600 mg/L B	75.3 ± 0.9 A	74.9 ± 1.0 A	1713 ± 34 B	1601 ± 26 B
1200 mg/L B	77.0 ± 1.3 A	76.4 ± 1.9 A	1815 ± 57 A	1680 ± 40 A
**Mepiquat chloride (MC)**				
Control	72.8 ± 0.9 B	71.9 ± 1.0 B	1583 ± 35 C	1481 ± 22 C
Squaring	76.6 ± 1.3 A	76.2 ± 1.9 A	1821 ± 49 A	1682 ± 36 A
Flowering	75.4 ± 0.7 A	75.1 ± 1.1 A	1723 ± 44 B	1607 ± 33 B
**Source of variation (Pr > F)**				
D	0.004	0.002	<0.001	<0.001
B	0.001	0.002	<0.001	<0.001
MC	0.004	0.004	<0.001	<0.001
D × B	0.808	0.917	*0*.*042*	*0*.*044*
D × MC	0.912	0.947	0.799	0.749
B × MC	0.809	0.956	*0*.*010*	*0*.*033*
D × B × MC	0.941	0.903	0.874	0.977

Values are mean ± SE of three replications. Means in a column followed by different letters are significantly different at P < 0.05. Interactions with P < 0.05 are italicized to indicate their significance.

**Figure 1:**
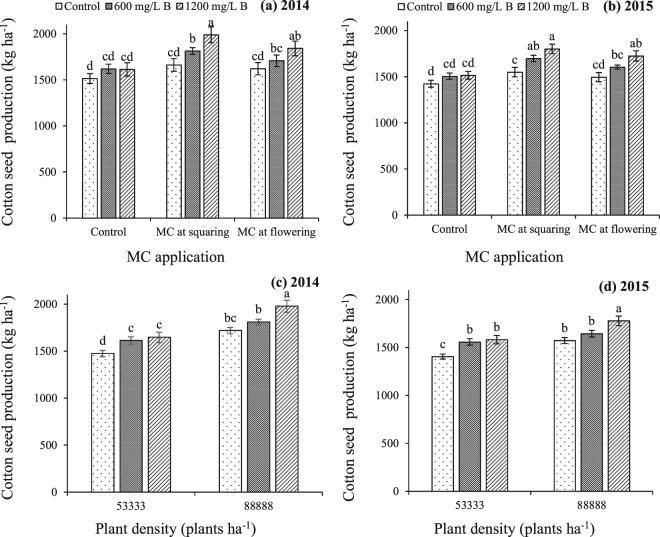
Cotton seed production as affected by interaction effects of (**a**,**b**) boron with mepiquat chloride and (**c**,**d**) boron with plant density. Bars are mean ± SE of three replications. Bars sharing same letter don’t differ significantly at P < 0.05.

Cotton seed production ha^−1^ was higher by 16.3% during 2014 and 9.9% during 2015 at higher plant density as compared to lower plant density (Table 1). Boron nutrition across both plant densities and MC treatments improved the cotton seed production, as compared to control (13.5 and 12.8% during 2014 and 2015, respectively) (Table 1). Mepiquat chloride across all plant density and B treatments significantly improved the seed production, as compared to control; with greatest increase caused by MC treatment at squaring stage (15% during 2014 and 13.6% during 2015) (Table 1). During both years significant interactions of B with MC and B with plant density were observed, pertaining to cotton seed production (Table 1). Cotton seed production was greatest when 1200 mg/L B was applied in combination with MC application at squaring stage (Fig. 1a,b). Likewise, B nutrition at 1200 mg/L at higher plant density led to the production of highest cotton seed (Fig. 1c,d).

**Figure 2:**
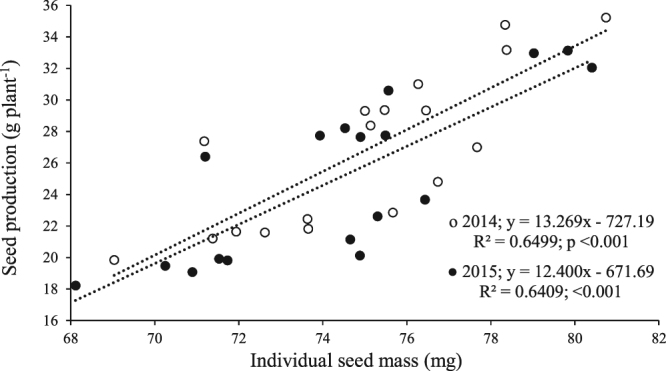
Relationship between individual seed mass (mg) and seed production (g plant^−1^) during 2014 and 2015. Coefficient of determination (R^2^), significance of coefficient (P < 0.05) and dependence of seed production (y) on individual seed mass (x) are given (n = 18).

A significant linear relationship between individual seed mass and seed production per plant occurred as affected by plant density, B nutrition and MC plant growth regulation, during both years. The linear relationship between seed mass and seed production per plant indicates that seed production varied uniformly by mean individual seed mass (Fig. 2).

### Seed nutrients reserves

Seed primary macronutrients (N, P and K) and micronutrients (B, Zn, Fe and Mn) reserves significantly differed between plant densities. Higher seed N (2.1 and 2.2%), P (5.1 and 8.2%), K (9.9 and 8.4%), B (19.9 and 20.5%), Zn (5.4 and 6.7%), Fe (4 and 3.6%) and Mn (8.6 and 7%) contents were produced at lower plant density as compared to higher plant density during 2014 and 2015, respectively (Tables 2 and 3). Significantly greater seed N (4.7 and 4.5%), P (8.3 and 12.1%), K (12.1 and 10.4%), B (31.6 and 36.4%), Zn (11.4 and 10%) and Fe (14.3 and 11.2%) reserves were observed in response to foliage B nutrition across MC and plant density treatments as compared to control, during 2014 and 2015, respectively. The P contents did not differ between 600 and 1200 mg/L B during 2015. However, Mn contents were significantly decreased (10.4 and 11%) with concomitant increase in B concentration during 2014 and 2015, respectively (Tables 2 and 3). Compared with control, MC led to increase in seed nutrients reserves; although no significant differences occurred between both treatment stages except K during 2015 and B during both years. Across B and plant density treatments the greatest enrichment of seed with N (3.8 and 4.2%), P (8.3 and 12.1%), K (12.2 and 11%), B (18.2 and 17.8%), Zn (9.2 and 8.2%) and Fe (3.4 and 4.9%) was caused by treatment of MC at squaring stage, during 2014 and 2015, respectively. Seed Mn contents were decreased (5.8 and 4.7%) by MC application while there was no difference between both stages of MC application (Tables 2 and 3).

**Table 2:** Cotton seed primary macronutrients reserves as influenced by plant density, foliar boron nutrition and mepiquat chloride application.

Treatments	Seed nitrogen (mg g^−1^ DW)		Seed phosphorus (mg g^−1^ DW)		Seed potassium (mg g^−1^ DW)	
	2014	2015	2014	2015	2014	2015
**Density (D) (plants ha** ^**−1**^ **)**						
53333	32.9 ± 0.23 A	32.1 ± 0.26 A	3.9 ± 0.04 A	3.71 ± 0.06 A	20.3 ± 0.37 A	19.0 ± 0.05 A
88888	32.2 ± 0.24 B	31.4 ± 0.22 B	3.7 ± 0.04 B	3.40 ± 0.05 B	18.3 ± 0.34 B	17.4 ± 0.02 B
**Boron nutrition (B)**						
Control	31.8 ± 0.27 B	31.0 ± 0.32 B	3.6 ± 0.05 C	3.3 ± 0.06 B	18.2 ± 0.48 C	17.3 ± 0.31 C
600 mg/L	32.5 ± 0.26 AB	31.9 ± 0.27 AB	3.8 ± 0.04 B	3.6 ± 0.07 A	19.2 ± 0.40 B	18.3 ± 0.32 B
1200 mg/L	33.3 ± 0.29 A	32.4 ± 0.25 A	3.9 ± 0.05 A	3.7 ± 0.08 A	20.4 ± 0.49 A	19.1 ± 0.33 A
**Mepiquat chloride (MC)**						
Control	31.9 ± 0.28 B	31.1 ± 0.25 B	3.6 ± 0.05 B	3.3 ± 0.06 B	18.1 ± 0.41 B	17.2 ± 0.29 C
Squaring	33.1 ± 0.27 A	32.4 ± 0.27 A	3.9 ± 0.05 A	3.7 ± 0.08 A	20.3 ± 0.47 A	19.1 ± 0.33 A
Flowering	32.6 ± 0.29 AB	31.8 ± 0.32 AB	3.8 ± 0.06 A	3.6 ± 0.07 A	19.5 ± 0.48 A	18.3 ± 0.32 B
**Source of variation (Pr > F)**						
D	0.025	0.034	<0.001	<0.001	<0.001	<0.001
B	0.002	0.004	<0.001	<0.001	<0.001	<0.001
MC	0.008	0.006	<0.001	<0.001	<0.001	<0.001
D × B	0.913	0.875	0.480	0.865	0.455	0.841
D × MC	0.996	0.984	0.754	0.780	0.887	0.947
B × MC	0.957	0.998	0.715	0.167	0.821	0.941
D × B × MC	0.834	0.986	0.316	0.388	0.461	0.974

Values are mean ± SE of three replications. Means in a column followed by different letters are significantly different at P < 0.05. Interactions with P < 0.05 are italicized to indicate their significance.

**Table 3:** Cotton seed micronutrients reserves as influenced by plant density, foliar boron nutrition and mepiquat chloride application.

Treatments	Seed boron (µg g^−1^ DW)		Seed zinc (µg g^−1^ DW)		Seed iron (µg g^−1^ DW)		Seed manganese (µg g^−1^ DW)	
	2014	2015	2014	2015	2014	2015	2014	2015
**Density (D) (plants ha** ^**−1**^ **)**								
53333	48.2 ± 1.1 A	41.5 ± 0.2 A	44.8 ± 0.56 A	44.7 ± 0.56 A	186.2 ± 2.35 A	170.7 ± 2.05 A	21.0 ± 0.30 A	21.4 ± 0.26 A
88888	38.6 ± 1.2 B	33.0 ± 0.2 B	42.4 ± 0.63 B	41.7 ± 0.53 B	178.8 ± 2.28 B	164.6 ± 2.12 B	19.2 ± 0.28 B	19.9 ± 0.31 B
**Boron nutrition (B)**								
Control	36.7 ± 1.30 C	30.8 ± 0.99 C	41.3 ± 0.60 C	41.1 ± 0.67 C	169.6 ± 1.84 C	158.6 ± 2.34 C	21.2 ± 0.38 A	21.8 ± 0.30 C
600 mg/L	45.2 ± 1.52 B	38.8 ± 1.37 B	43.4 ± 0.55 B	43.4 ± 0.69 B	184.1 ± 1.74 B	168.1 ± 1.58 B	20.1 ± 0.31 B	20.7 ± 0.31 B
1200 mg/L	48.3 ± 1.44 A	42.0 ± 1.38 A	46.0 ± 0.75 A	45.2 ± 0.58 A	193.8 ± 1.81 A	176.3 ± 2.07 A	19.0 ± 0.38 C	19.4 ± 0.33 A
**Mepiquat chloride (MC)**								
Control	39.5 ± 1.55 C	33.8 ± 1.26 C	41.4 ± 0.58 B	41.4 ± 0.67 B	178.9 ± 2.68 B	162.6 ± 2.43 B	20.7 ± 0.37 A	21.2 ± 0.39 A
Squaring	46.7 ± 1.86 A	39.8 ± 1.75 A	45.2 ± 0.71 A	44.8 ± 0.70 A	185.0 ± 2.91 A	170.6 ± 3.01 A	19.5 ± 0.40 B	20.2 ± 0.35 B
Flowering	44.1 ± 1.69 B	38.1 ± 1.70 B	44.2 ± 0.76 A	43.4 ± 0.69 A	183.6 ± 3.19 AB	169.8 ± 2.07 A	20.1 ± 0.43 AB	20.5 ± 0.40 AB
**Source of variation (Pr > F)**								
D	<0.001	<0.001	<0.001	<0.001	<0.001	0.002	<0.001	<0.001
B	<0.001	<0.001	<0.001	<0.001	<0.001	<0.001	0.001	<0.001
MC	<0.001	<0.001	<0.001	<0.001	0.001	0.001	0.032	0.032
D × B	*0*.*024*	*0*.*036*	0.738	0.526	0.645	0.519	0.828	0.838
D × MC	0.128	*0*.*034*	0.999	0.967	0.764	0.653	0.746	0.744
B × MC	*0*.*036*	*0*.*002*	0.433	0.754	0.973	0.238	0.985	0.995
D × B × MC	0.649	0.134	0.530	0.835	0.874	0.940	0.952	0.841

Values are mean ± SE of three replications. Means in a column followed by different letters are significantly different at P < 0.05. Interactions with P < 0.05 are italicized to indicate their significance.

**Figure 3:**
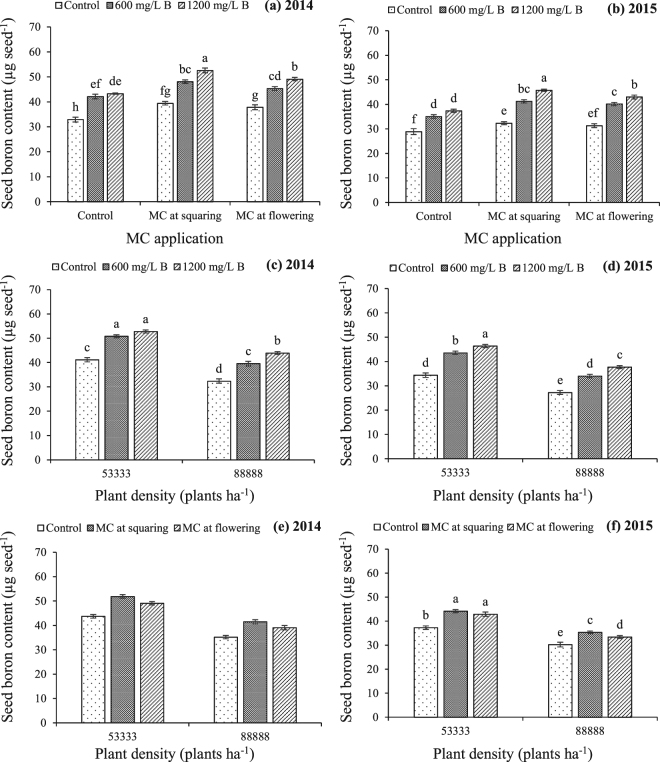
Cotton seed boron content as affected by interaction effects of (**a**,**b**) boron with mepiquat chloride, (**c**,**d**) boron with plant density and (**e**,**f**) mepiquat chloride with plant density. Bars are mean ± SE of three replications. Bars sharing same letter don’t differ significantly at P < 0.05.

Interactions of plant density, B and MC were non-significant for seed nutrients reserves (N, P, K, Zn, Fe and Mn); except B for which the interaction effects of B with MC and B with plant density were significant, during both years while interaction of MC with plant density was significant during 2015 (Tables 2 and 3). Seed B contents were substantially increased by application of 1200 mg/L B plus MC application at squaring stage (Fig. 3a,b). The B application at 1200 mg/L at lower plant density resulted in significantly higher seed B reserves (Fig. 3c,d). Similarly, MC application at squaring stage on lower plant density caused the greatest improvement in seed B contents during 2015 (Fig. 3f).

Seed nutrients contents (N, P, K, B, Zn and Fe) and individual seed mass were positively correlated in response to plant density, foliar B nutrition and MC growth regulation, during both years (Table 4). This positive relationship manifested that changes in seed mass and macro- and micronutrients contents were relatively uniform across treatments (Table 4).

**Table 4:** Pearson’s coefficients of correlation between individual seed mass and seed nutrients (N, P, K, B, Zn, Fe and Mn) contents as influenced by plant density, foliar boron nutrition and mepiquat chloride application (n = 18).

Variables	Individual seed mass (mg)	
	2014	2015
Seed nitrogen content (mg g^−1^ DW)	0.9146^**^	0.9634^**^
Seed phosphorus content (mg g^−1^ DW)	0.9456^**^	0.9357^**^
Seed potassium content (mg g^−1^ DW)	0.9372^**^	0.9652^**^
Seed boron content (mg g^−1^ DW)	0.9428^**^	0.9449^**^
Seed zinc content (mg g^−1^ DW)	0.9270^**^	0.9242^**^
Seed iron content (mg g^−1^ DW)	0.8686^**^	0.8713^**^
Seed manganese content (mg g^−1^ DW)	−0.3183^NS^	−0.3345^NS^

^**^P < 0·01; ^*^P < 0·05; ^NS^non-significant.

### Emergence and seedling vigour

Final emergence and seedling vigour index were significantly greater from seed produced at lower plant density with concomitant decrease in mean emergence time. Higher final emergence (6.3 and 5.9% during 2014 and 2015, respectively) occurred at lower plant density than higher plant density (Table 5). Foliage B nutrition of maternal cotton plants significantly improved the emergence and seedling vigour index. Final emergence was higher by 7.2 and 8.9 percentages during 2014 and 2015, respectively, by 1200 mg/L B nutrition, as compared to control. Moreover, compared with control, same B nutrition rate encountered a decrease in mean emergence time by 6.5 and 4.3% during 2014 and 2015, respectively (Table 5). Likewise, MC application at squaring stage on maternal plants was superior in improving the final emergence (6.7 and 7.8% during 2014 and 2015, respectively) and seedling vigour index (20.9 and 22.7% during 2014 and 2015, respectively) (Table 5).

**Table 5:** Emergence, emergence time and seedling vigour index of cotton from seed produced by plants in response to plant density, foliar boron nutrition and mepiquat chloride application.

Treatments	Final emergence (%)		Mean emergence time (days)		Seedling vigour index	
	2014	2015	2014	2015	2014	2015
**Density (D) (plants ha** ^**−1**^ **)**						
53333	66 ± 2 A	71 ± 2 A	4.6 ± 0.1 A	4.9 ± 0.0 B	1860 ± 49 A	1808 ± 48 A
88888	60 ± 1 B	65 ± 2 B	4.6 ± 0.2 A	5.0 ± 0.1 A	1576 ± 39 B	1562 ± 39 B
**Boron nutrition (B)**						
Control	59 ± 1 B	63 ± 2 B	4.8 ± 0.0 A	5.1 ± 0.1 A	1526 ± 40 C	1484 ± 37 C
600 mg/L	63 ± 3 AB	69 ± 2 A	4.5 ± 0.1 B	4.9 ± 0.1 AB	1735 ± 53 B	1726 ± 55 B
1200 mg/L	67 ± 2 A	72 ± 4 A	4.5 ± 0.0 B	4.9 ± 0.0 B	1893 ± 64 A	1845 ± 56 A
**Mepiquat chloride (MC)**						
Control	59 ± 2 B	64 ± 2 B	4.7 ± 0.2 A	5.0 ± 0.1 A	1537 ± 40 C	1509 ± 41 C
Squaring	66 ± 4 A	72 ± 4 A	4.5 ± 0.1 B	4.9 ± 0.0 A	1858 ± 62 A	1852 ± 57 A
Flowering	64 ± 2 AB	68 ± 3 AB	4.5 ± 0.1 B	4.9 ± 0.1 A	1759 ± 63 B	1694 ± 56 B
**Source of variation (Pr > F)**						
D	<0.001	0.004	0.487	0.014	<0.001	<0.001
B	0.001	0.002	<0.001	0.035	<0.001	<0.001
MC	0.003	0.008	<0.001	0.173	<0.001	<0.001
D × B	0.940	0.963	0.166	0.848	0.545	0.114
D × MC	0.780	0.768	0.856	0.804	0.561	0.497
B × MC	0.908	0.921	0.600	0.783	*0*.*009*	*0*.*039*
D × B × MC	0.908	0.961	0.976	0.991	0.229	0.093

Values are mean ± SE of three replications. Means in a column followed by different letters are significantly different at P < 0.05. Interactions with P < 0.05 are italicized to indicate their significance.

**Figure 4:**
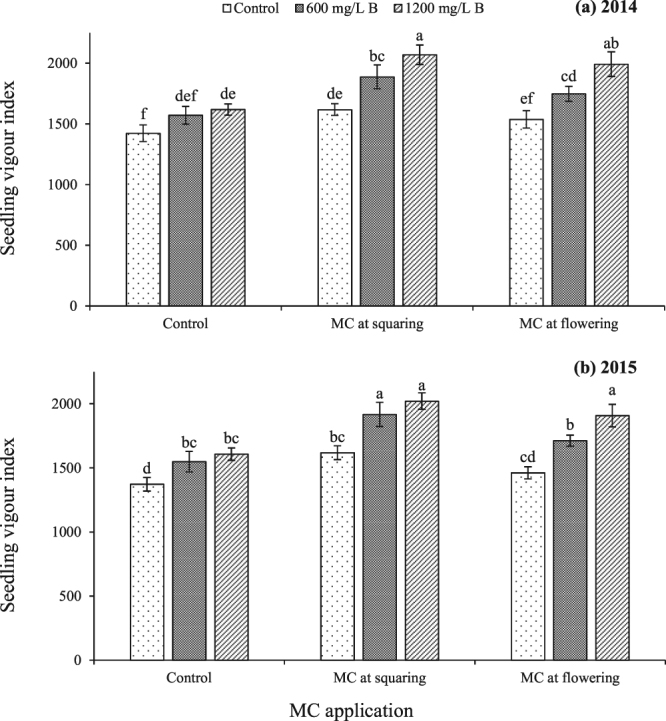
Seedling vigour index of cotton seedlings from seed produced by plants in response to interaction effect of foliar boron nutrition with mepiquat chloride application. Bars are mean ± SE of three replications. Bars sharing same letter don’t differ significantly at P < 0.05.

**Figure 5:**
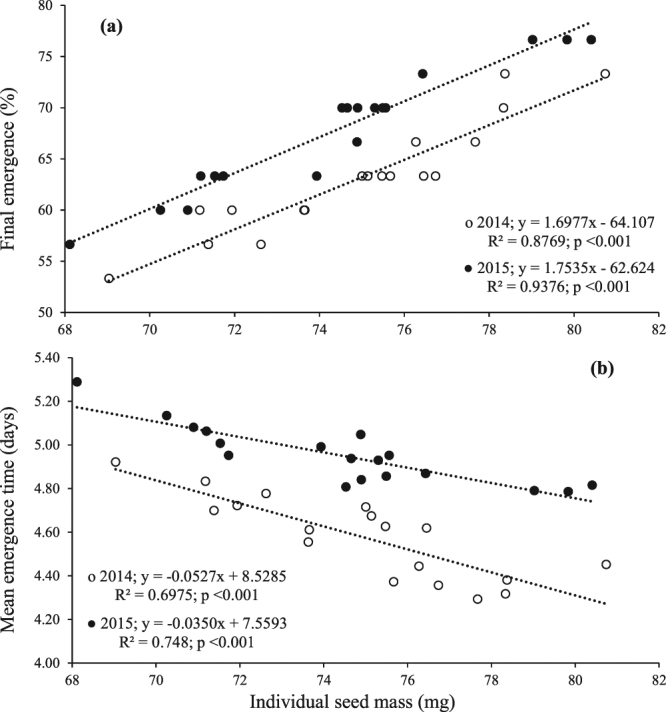
Relationship between (**a**) individual seed mass (mg) (mean) and final emergence (%), and (**b**) seed mass (mg) and mean emergence time (days) during 2014 and 2015. Coefficients of determination (R^2^), significance of coefficients (P < 0.05) and dependence of (**a**) final mergence percentage (y) and (**b**) mean emergence time (y) on individual seed mass (x) are given (n = 18).

The interactions of plant density, B nutrition and MC application were non-significant for final emergence and mean emergence time. However, the interaction effect of B with MC was significant for seedling vigour index during both years (Table 5). Substantially greater seedling vigour index was perceived by 1200 mg/L B plus MC application at squaring stage, during both years (Fig. 4a,b). Linear regression manifested a linear increase in final emergence with seed mass (Fig. 5a). However, there was a negative linear relation between seed mass and mean emergence time indicating a linear decrease in mean emergence time with increase in seed mass (Fig. 5b).

### Offspring seedling growth

**Figure 6:**
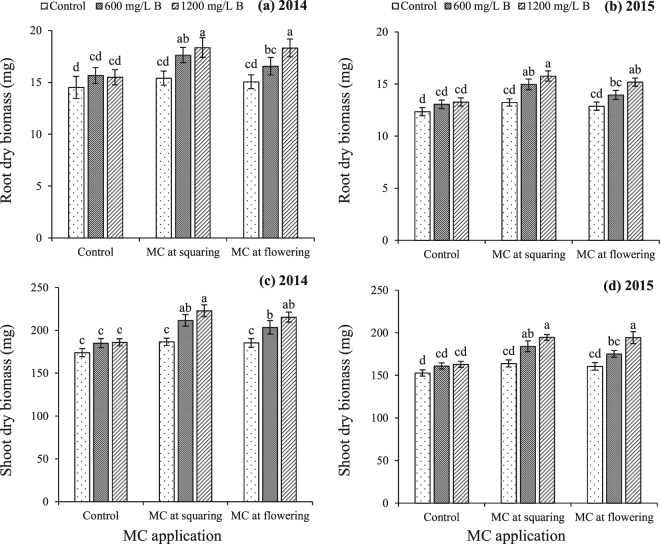
Seedling traits [(**a**,**b**) root dry biomass and (**c**,**d**) shoot dry biomass] of cotton from seed produced by plants in response to interaction effect of foliar boron nutrition and mepiquat chloride application. Bars are mean ± SE of three replications. Bars sharing same letter don’t differ significantly at P < 0.05.

**Figure 7:**
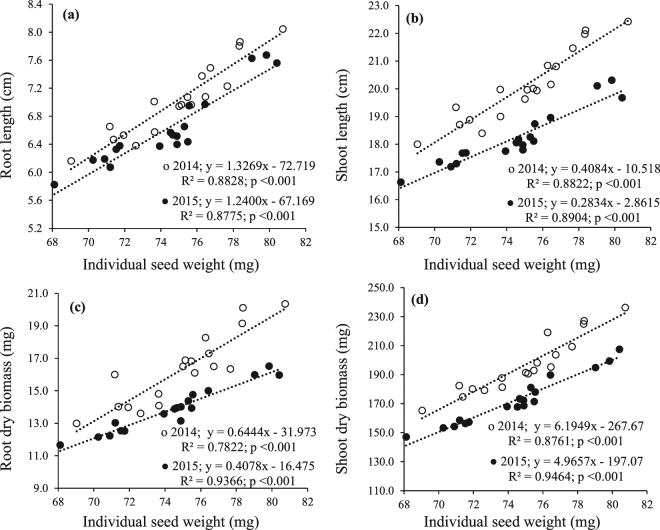
Relationship between (**a**) individual seed mass (mg) and root length (cm), (**b**) individual seed mass (mg) and shoot length (cm), (**c**) individual seed mass (mg) and root dry biomass (mg), and (**d**) individual seed mass (mg) and shoot dry biomass (mg) during 2014 and 2015. Coefficients of determination (R^2^), significance of coefficients (P < 0.05) and dependence of (**a**) root length (y), (**b**) shoot length (y), (**c**) root dry biomass (y) and (**d**) shoot dry biomass (y) on individual seed mass (x) are given (n = 18).

Seedling growth traits (root length, shoot length, root dry biomass and shoot dry biomass) of cotton in succeeding generation were significantly greater at lower plant density as compared to higher plant density (Table 6). Boron treatment on cotton plants effectively improved the offspring seedling growth traits of cotton and the effect was greater at 1200 mg/L B than lower concentration (600 mg/L) and control (Table 6). Growth regulation by MC manifested a similar significant improvement in seedling growth traits as compared to control, during both years (Table 6). The interactions for root length and shoot length were non-significant; however, interactive effect of B with MC on root and shoot dry biomass was significant, during both years (Table 6). The greatest root and shoot dry biomass accumulation occurred by B nutrition at 1200 mg/L concentration in combination with MC treatment at squaring stage (Fig. 6c,d). There was a significant positive linear relationship between seedling growth traits and seed mass indicating that seedling growth traits were increased uniformly with increase in seed mass (Fig. 7a–d).

**Table 6:** Seedling traits (root length, shoot length, root dry biomass and shoot dry biomass) of cotton from seed produced by plants in response to plant density, foliar boron nutrition and mepiquat chloride application.

Treatments	Root length (cm)		Shoot length (cm)		Root dry biomass (mg)		Shoot dry biomass (mg)	
	2014	2015	2014	2015	2014	2015	2014	2015
**Density (D) (plants ha** ^**−1**^ **)**								
53333	7.3 ± 0.4 A	6.9 ± 0. A	20.72 ± 0.8 A	18.7 ± 0.5 A	17.9 ± 1.0 A	14.6 ± 0.4 A	206.7 ± 3.9 A	179.4 ± 3.4 A
88888	6.8 ± 0.1 B	6.4 ± 0.1 B	19.47 ± 0.3 B	17.8 ± 0.3 B	14.7 ± 0.3 B	13.1 ± 0.2 B	186.6 ± 3.1 B	165.0 ± 3.0 B
**Boron nutrition (B)**								
Control	6.6 ± 0.2 B	6.2 ± 0.1 B	19.05 ± 0.3 B	17.5 ± 0.2 B	14.9 ± 0.6 C	12.8 ± 0.2 C	181.9 ± 2.9 C	159.2 ± 2.5 C
600 mg/L	7.1 ± 0.1 A	6.7 ± 0.2 A	20.27 ± 0.4 A	18.4 ± 0.8 AB	16.6 ± 0.7 B	13.9 ± 0.3 B	200.0 ± 4.5 B	173.5 ± 3.5 B
1200 mg/L	7.4 ± 0.4 A	6.9 ± 0.2 A	20.97 ± 0.9 A	18.8 ± 0.3 A	17.4 ± 1.1 A	14.7 ± 0.3 A	208.1 ± 5.0 A	183.9 ± 4.5 A
**Mepiquat chloride (MC)**								
Control	6.7 ± 0.2 B	6.2 ± 0.1 B	19.20 ± 0.3 B	17.5 ± 0.3 B	15.2 ± 0.4 B	12.9 ± 0.2 C	181.6 ± 2.9 B	158.9 ± 2.2 B
Squaring	7.3 ± 0.4 A	6.9 ± 0.2 A	20.79 ± 0.5 A	18.9 ± 0.7 A	17.1 ± 0.7 A	14.7 ± 0.4 A	207.0 ± 4.9 A	180.9 ± 4.0 A
Flowering	7.2 ± 0.2 A	6.7 ± 0.1 A	20.30 ± 0.4 AB	18.3 ± 0.3 AB	16.6 ± 0.5 A	14.0 ± 0.3 B	201.4 ± 4.6 A	176.8 ± 4.4 A
**Source of variation (Pr > F)**								
D	<0.001	<0.001	0.002	0.022	<0.001	<0.001	<0.001	<0.001
B	<0.001	<0.001	0.001	0.019	<0.001	<0.001	<0.001	<0.001
MC	0.001	<0.001	0.005	0.010	<0.001	<0.001	<0.001	<0.001
D × B	0.965	0.119	0.918	0.738	0.553	0.671	0.114	0.912
D × MC	0.635	0.169	0.966	0.745	0.820	0.934	0.417	0.793
B × MC	0.556	0.372	0.574	0.925	*0*.*005*	*0*.*029*	*0*.*021*	*0*.*012*
D × B × MC	0.964	0.192	0.966	0.902	0.786	0.989	0.515	0.235

Values are mean ± SE of three replications. Means in a column followed by different letters are significantly different at P < 0.05. Interactions with P < 0.05 are italicized to indicate their significance.

## Discussion

The results of this study have revealed new information that maternal plant density, foliage B nutrition and MC growth regulation influence the offspring performance in terms of emergence and seedling growth plasticity in annual cultivated cotton.

### Seed nutrients reserves, seed mass and production

Individual seed mass of cotton was decreased at higher plant density while increased by foliage B nutrition and MC growth regulation (Table 1). This might be attributed to variation in nutrient availability and uptake in response to maternal environmental variables as indicated by differential seed nutrients reserves (Tables 2 and 3). Cotton seed nutrients reserves were increased at higher plant density relative to lower plant density. However, in comparison with control, B and MC enhanced the seed nutrients contents at both plant densities except Mn (Tables 2 and 3). This variation in cotton seed nutrients reserves might be explained on the basis of root growth which is necessary for nutrient uptake^48^. The root growth is depressed at higher plant density due to competition^20^ while increased by B^49^ and MC^47^ influencing the nutrient dynamics within plants. Moreover, B and MC alters the ability of plants for nutrient uptake and translocation through improved nutrient metabolism and assimilation^24,50–52^. However, decreased seed Mn content by B may be attributed to their antagonistic effect with each other that led to decreased uptake and translocation^53^. Previous studies have reported similar results that plant and seed nutrients contents decreased at high plant density^15,20^, while increased in response to B nutrition^24,26,27^ and MC application^6,14^.

Although little attention has been given to the relationship between seed nutrients contents and seed size. In this study, positive linear relationship between seed nutrients contents (N, P, K, B, Zn and Fe) and seed mass (Table 4) manifested that large sized seeds were better provisioned due to greater availability of nutrients to developing seeds. Previous studies have reported that seed provisioning is associated with nutrient availability which may be influenced due to soil nutrient gradient^2^, competition among plants for available resources^4^ and/or nutrient enrichment^1^. Plants often growing in nutrient rich environments produce large sized seeds containing greater nutrient contents^17,54^. In this study, the strength of relationship between concentration of nutrients and seed mass (Table 4) was strong enough to indicate that bigger seeds contained greater nutrients contents (mg per seed) than the smaller seeds because of higher nutrient concentration in response to maternal ecological factors. It is also evident from the calculations from seed mass and nutrients concentrations (Tables 1–3) that the seeds produced at lower plant density with B nutrition and MC growth regulation had greater nutrients reserves than seeds produced by plants at higher plant density and without B nutrition and MC growth regulation. Similarly, Balestri *et al*.^12^ found higher C, N and P contents in bigger seeds of *Posidonia oceanica* than smaller ones.

In this study, individual seed mass was linearly related to seed production per plant of cotton (Fig. 2). Therefore, although seed production per hectare was increased (Table 1) but seed production per plant was decreased with increase in plant density due to decreased individual seed mass. However, seed production per plant and per hectare was increased by exogenous application of B and MC which was due to linear increase in seed mass (Fig. 2; Table 1). Similarly, N and P enrichment of maternal plants of *Stipa krylovii* and *Artemisia frigida* produced heavier seeds in temperate steppe ecosystem. Furthermore, a significant positive relationship existed between seed mass and seed production^1^.

### Emergence and emergence timing

There was a positive linear relationship of seed mass with final emergence and emergence timing of cotton in response to plant density, B nutrition and MC growth regulation (Fig. 5a,b). The results of this study are consistent with the fact that heavier seeds germinate rapidly and gives better seed germination^55^. This indicates that bigger seeds having greater seed nutrients reserves are advantageous pertaining to emergence and emergence timing, and have more time for the seedling establishment. Moreover, lower seedling vigour index of seed from plants at higher plant density and higher seedling vigour index of seed from B and MC treated plants (Fig. 4a,b; Table 5) indicates that higher amounts of seed nutrients reserves in large sized seeds enhanced the emergence and emergence timing, and had better seedling establishment than smaller seeds with less nutrients reserves. Our results are consistent with previous reports that seed mass has relationship with germination and seedling vigour. van Mölken *et al*.^3^ observed that higher germination percentage was produced by heavier seeds of *Tragopogon pratensis* subsp. *pratensis* from Asteraceae family. Ninh *et al*.^56^ reported positive relationship between seed size and germination percentage of *Angelica acutiloba*. Yanlong *et al*.^55^ reported that large sized seeds of *Ligularia virgaurea* had higher germination, germination rate and seedling vigour under both light and shade conditions.

### Offspring growth plasticity

The seedling growth of offspring significantly differed by the influence of plant density, B nutrition and MC growth regulation. The seeds from plants at higher plant density produced seedlings with lesser root and shoot length, and root and shoot dry biomass. On the other hand, seedlings from seeds produced by B and MC treated plants had greater root and shoot length as well as dry biomass, as compared to untreated control (Fig. 6a–d; Table 6). This growth plasticity can be explained by the fact that large sized seeds with greater nutrients reserves produce vigorous seedlings due to early head start and greater energy available^2^. It was also evident from significant linear relationship between seedling growth traits and seed mass (Fig. 7a–d) that bigger seeds produced more successful seedlings with more competitive ability than smaller seeds. The vigorous seedlings produced from bigger seeds with greater nutrients reserves have more competitive ability over seedlings from small seeds with less reserves^57^ which is advantageous especially in agro-ecosystems where higher competitive ability of crop plants is beneficial in improving the system productivity with minimum inclusion of external inputs.

Results of this study suggest that offspring growth performance is affected more by seed nutrients reserves rather than seed phenotypic traits. This is because firstly, the seed phenotypic traits especially seed size is affected by nutrient availability^2,4,17,57^, as indicated by positive relationship between seed nutrients reserves and individual seed mass in this study. Secondly, increase in seed size (mass) may be the result of increased mass of seed structures (seed coat, awns etc.) rather than seed embryo or endosperm^1,58^. Moreover, in this study a significant negative relationship between seed mass and mean emergence time (Fig. 5b) indicated that the seedlings from larger seeds started early vigorous growth and still had more time to develop due to greater amounts of nutrients reserves. Yanlong *et al*.^55^ reported that seedlings of *L*. *virgaurea* produced from larger seeds had greater dry biomass. Similarly Kolodziejek^2^ observed that maternal enrichment of *S*. *krylovii* and *A*. *frigida* with N and P produced heavier seeds that led to vigorous seedlings with higher initial growth.

In summary, plant density, B nutrition and MC plant growth regulation significantly affected the seed mass of cotton and seed nutrients reserves (N, P, K, B, Zn and Fe). The positive linear relationship between seed nutrients reserves and seed mass indicated that nutrient availability and/or uptake influence the seed phenotypic traits in response to maternal ecological factors. The variation in seed mass ultimately influenced the cotton seed production, offspring emergence and seedling growth plasticity. The results suggest that lower maternal plant density, B nutrition and MC growth regulation results in higher emergence and offspring seedling growth due to higher seed mass and nutrients reserves. This improved emergence and competitive ability of offspring seedlings of cotton through better management practices could be exploited to improve the agro-ecosystem productivity while reducing the input cost (e.g. seed, fertilizer, herbicide etc.).

## Methods

### Field experimental site

A 2-years field experiment was conducted at Agronomic Research Area, University of Agriculture, Faisalabad (31.25°N, 73.06°E and 183 masl), Pakistan, during 2014 and 2015. Soil samples were collected prior to sowing the crop using auger from a depth of 0–30 cm from different locations of field to determine the physico-chemical properties of soil, during both years. The soil was alkaline-calcareous, B deficient and sandy loam in nature with high pH (8.2, 8.1), calcium carbonate (5.20, 5.11%) and electrical conductivity (1.78, 1.72 dS m^−1^), while lower in organic matter (1.12, 0.97%). The soil was medium in nutrient status with lower total N (0.050, 0.049%), available P (7.09, 6.91 ppm), exchangeable K (259, 248 ppm), B (0.47, 0.49 ppm), Zn (1.70, 1.50 ppm) and Fe (5.21, 5.10 ppm) during both years, respectively. The soil of experimental site belongs to Lyallpur soil series (aridisol fine silty, mixed, hyperthermic Ustalfic), Haplarged in the USDA classification and Haplic Yermosols in the FAO classification. The meteorological data during both experimental seasons are given in Table 7.

**Table 7:** Meteorological conditions during cotton growing seasons of field experiment.

Month	Total rainfall (mm)		Relative humidity (%)		Temperature (°C)						Sunshine (h)	
					Monthly maximum		Monthly minimum		Daily mean			
	2014	2015	2014	2015	2014	2015	2014	2015	2014	2015	2014	2015
May	41.2	17.0	33.2	27.5	36.6	38.7	23.7	24.9	30.1	31.8	10.4	10.4
June	7.1	11.6	33.5	39.0	40.9	38.0	28.1	25.6	34.5	31.8	9.4	9.4
July	57.5	128.0	53.9	61.1	37.0	34.9	28.0	27.0	32.5	31.0	9.0	5.1
August	4.8	48.4	52.7	60.4	37.1	35.9	27.3	26.7	32.2	31.3	9.1	7.0
September	140.2	75.2	61.2	51.6	33.9	35.4	24.5	24.4	29.2	29.9	7.7	8.2
October	3.6	14.5	54.6	52.9	31.3	32.2	19.1	19.1	25.2	25.4	7.8	8.3
November	10.0	8.8	61.7	61.5	26.3	27.1	11.5	12.1	18.9	19.6	7.6	6.6

Source: Agro-meteorology Cell, Department of Crop Physiology, University of Agriculture, Faisalabad, Pakistan.

### Field experimental design

This study was accomplished in two phases. In first phase, 2-year field experiment was conducted. The experiment was carried out using randomized complete block design with three-factor factorial arrangement including three replications. Net plot size was 6 m × 3 m. The treatment variables were two plant densities [lower plant density (53333 plants ha^−1^) and higher plant density (88888 plants ha^−1^)], foliage applied B (0, 600 and 1200 mg/L B) and single foliar applications of MC solution with 70 mg/L concentration at two different growth stages *viz*. squaring and flowering. Control plants received water spray. The B was foliage applied five weeks after sowing using boric acid (17% B); while, MC [98% SP from Henan Haoyuhang Economic and Trade Co., Ltd] was foliar applied using a Knapsack hand sprayer at a pressure of 207 kPa. The spray volume for MC application at squaring was 300 L ha^−1^ while for B and MC application at flowering stage was 350 L ha^−1^.

### Field management

Cotton cultivar MNH-886 (Containing Bt gene, developed by Central Cotton Research Institute, Multan, Pakistan) was sown on May 22^nd^ during 2014 and May 25^th^ during 2015. Prior to sowing, the scarification treatment of seed was carried out with commercial sulfuric acid (1:10 ratio of seed and acid) and treated with fungicide (dynasty CST 125 FS @ 3 g kg^−1^ seed). The sowing was carried out at beds using dibbler and keeping the inter row spacing of 75 cm while intra row spacing was varied according to the treatments i.e. 25 and 15 cm to maintain plant densities of 53333 and 88888 plants ha^−1^, respectively. Seed rates of 15 and 25 kg ha^−1^ was used for attaining plant densities of 53333 and 88888 plants ha^−1^, respectively and 2–3 seeds were sown per hill. Thinning was carried out 25 DAS to keep the desired plant densities. Fertilization was carried out at the rate of 200-120-110 kg NPK ha^−1^ using urea (46% N), diammonium phosphate (18% N: 46% P_2_O_5_) and sulfate of potash (50% K_2_O), during both years. All P, K and 1/3^rd^ N was applied at sowing; while, remaining N was applied at squaring and boll formation stages in equal splits. The crop was irrigated eight times during 2014 and five times during 2015 besides irrigation applied at sowing keeping the crop and weather conditions in consideration. During second year, occurrence of heavy rainfall decreased the irrigation requirements (Table 7). Plant protection measures were carried out according to the local recommendations in order to keep the weeds, insect pests and diseases below economic threshold level.

### Measurements of seed mass and seed nutrients reserves

The crop was harvested in two manual pickings. The seed cotton obtained from all pickings was mixed and ginned using roller type, hand-fed laboratory gin. Cotton seed production (kg ha^−1^) was determined after ginning. Individual cotton seed mass was determined by taking the mean mass of 100 seeds collected randomly after ginning. The delinted seed samples were assayed to determine the concentration of macro and micronutrients. The delinted seed samples were dried under sun and electric oven at 70 °C for 24 h, ground to powder form (Cyclotec 1093 Sample Mill, Sweden) to pass through a 30-mesh screen. The samples were digested using sulfuric acid and catalyst mixture and the N was determined by Kjeldhal distillation method as described by Estefan *et al*.^59^. The P was analyzed by ammonium-vanadomolybdate colorimetric method at 410 nm using the same extract^59^. For determination of K and micronutrients *viz*. Zn, Fe and Mn the samples were soaked over-nightly in di-acid mixture and digested on block digester. The K was determined by flame photometer using the method of Chapman and Pratt^60^; while, Zn, Fe and Mn were assayed using the atomic absorption spectrometer according to the method described by Estefan *et al*.^59^. Boron was determined by dry ashing the ground seed material in muffle furnace at 550 °C for 6 h^60^. The ash was taken in 0.36N H_2_SO_4_ and the B concentration was determined by spectrophotometer at 420 nm wavelength using azomethine-H colorimetric method^61^. The contents of seed macronutrients (N, P and K) was expressed as mg g^−1^ dry weight (DW) and micronutrients (B, Zn, Fe and Mn) as µg g^−1^ DW.

### Soil bioassay

In second phase, the seed collected from maternal plants was subjected to soil bioassay to assess the emergence and offspring plasticity in seedling growth. The bioassay was not carried out immediately after collection of seed, instead seeds were stored at room temperature (25 °C) to avoid effects of dormancy and poor seed germination. The bioassay was performed in subsequent year every time after collection of seed i.e. 2015 and 2016 (however, hereafter we shall call the years 2015 and 2016 as 2014 and 2015, respectively, to avoid confusion). The experiment was conducted by using completely randomized design with factorial arrangement and three replications. Ten seeds were sown in soil filled pots (25 cm diameter and 20 cm height) following scarification treatment with sulfuric acid (1:10 ratio of seed and acid). At the start 150 mL of deionized water was applied to each pot and then according to the requirement. The experiments were observed for 30 days. The average maximum temperature was 31.1 and 33.0 °C, and minimum temperature was 19.3 and 19.7 °C during first and second year, respectively, during the period of experiment.

### Measurements of emergence and seedling growth

The seedlings emerged were counted daily up to the end of experiment as per method of Association of Official Seed Analysts^62^. Final emergence percentage was calculated by using following the formula;

1 $$Final\,emergence({\rm{ \% }})=\,\frac{No.\,of\,seedlings\,emerged}{Total\,number\,of\,seeds}\times 100$$

Equation of Ellis and Roberts^63^ was used to calculate the mean emergence time;

2 $$Mean\,emergence\,time\,(days)=\,\frac{\sum Dn}{N}$$

where, *n* is the number of emerged seedlings on day *D* and *N* is the total number of germinated/emerged seeds.

At the end of experiment, seedlings were uprooted and their shoot and root lengths were measured. Shoots and roots were separated and dried in oven at 70 °C till constant weight to measure the dry biomass accumulation. Seedling vigour index (SVI) was calculated using the equation of Abdul-Baki and Anderson^64^:

3 $$SVI=Final\,emergence\,({\rm{ \% }})\times Seedling\,length\,(cm)$$

### Statistical analyses

Rainfall, air temperature and accumulation of heat units were different in 2014 and 2015 from May through November at field experimental site, therefore data of each year was analyzed separately. All the data were analyzed using the Univariate General Linear Models (GLM) procedure of SPSS 19. The plant density, B and MC were entered as fixed factors while replication (block) was entered as random factor. The treatments’ means were compared by using the Tukey’s honest significance difference (HSD) test at 5% probability. Pearson’s coefficients were calculated to determine the correlation between seed mass and seed nutrients contents (N, P, K, B, Zn, Fe and Mn) to test the effect of plant density, B nutrition and MC growth regulation on seed nutrients and seed mass. Linear regression analyses were performed to determine the quantitative relationship of individual seed mass with seed production, final emergence, mean emergence time and seedling growth traits.
