# Author Correction: Effect of Plant Density, Boron Nutrition and Growth Regulation on Seed Mass, Emergence and Offspring Growth Plasticity in Cotton

**DOI:** 10.1038/s41598-018-28825-9

**Published:** 2018-07-06

**Authors:** Ali Zohaib, Tahira Tabassum, Abdul Jabbar, Shakeel Ahmad Anjum, Tasawer Abbas, Azhar Mehmood, Sohail Irshad, Muhammad Kashif, Mohsin Nawaz, Naila Farooq, Irfan Rasool Nasir, Tassadduq Rasool, Mubashar Nadeem, Riaz Ahmad

**Affiliations:** 1 Department of Agronomy, University of Agriculture, Faisalabad, 38040 Pakistan; 2 Department of Agronomy, College of Agriculture, University of Sargodha, Sargodha, 40100 Pakistan; 3 Agronomy Department, University College of Agriculture and Environmental Sciences, The Islamia University of Bahawalpur, Bahawalpur, 63100 Pakistan; 4 Agricultural Training Institute, Rahim Yar Khan, 64200 Pakistan; 5 Department of Botany, University of Agriculture, Faisalabad, 38040 Pakistan; 6 Institute of Tropical Agriculture and Forestry, Hainan University, Haikou, 570228 P.R. China; 7 Institute of Soil and Environmental Sciences, University of Agriculture, Faisalabad, 38040 Pakistan; 8 Adaptive Research Farm, Dera Ghazi Khan, 32200 Pakistan

Correction to: *Scientific Reports* 10.1038/s41598-018-26308-5, published online 21 May 2018

The original version of this Article omitted an affiliation for Mohsin Nawaz. The correct affiliations for Mohsin Nawaz are listed below:

Department of Agronomy, University of Agriculture, Faisalabad, 38040, Pakistan

Institute of Tropical Agriculture and Forestry, Hainan University, Haikou, 570228, P.R. China

This has now been corrected in the HTML and PDF versions of this Article.

